# The relationship between physical exercise and mobile phone addiction among Chinese college students: Testing mediation and moderation effects

**DOI:** 10.3389/fpsyg.2022.1000109

**Published:** 2022-10-03

**Authors:** Miaolin Zeng, Siyu Chen, Xiangyi Zhou, Jincheng Zhang, Xin Chen, Jingquan Sun

**Affiliations:** ^1^Institute of Sports Science, Sichuan University, Chengdu, China; ^2^Department of Sport and Health Sciences, Technical University of Munich, Munich, Germany; ^3^School of Physical Education, Sichuan University, Chengdu, China

**Keywords:** physical exercise, Mobile phone addiction, college students, self-control, rumination, psychological distress, loneliness

## Abstract

**Background:**

During the COVID-19 pandemic, suspensions of activities and long periods of self-isolation led to a sharp increase in excessive use of mobile phones, which sparked public concern about mobile phone addiction (MPA). In recent years, more and more attention has been paid to physical exercise as a protective effect of MPA. However, more studies are needed to reveal this relationship and the exact mechanisms, based on which this study tested the mediating and moderating roles of self-control, rumination, psychological distress, and loneliness between physical exercise and MPA.

**Methods:**

In this cross-sectional study, primary data was collected by questionnaire from 1,843 college students (19.75 ± 1.3) from five universities in Sichuan Province in Mainland China. Mobile Phone Addiction Tendency Scale (MPATS), Physical Activity Rating Scale-3 (PARS-3), Self-Control Scale (SCS), Ruminative Response Scale (RRS), Depression Anxiety Stress Scale-21 (DASS-21), and UCLA Loneliness Scale (UCLA-20) were investigated. The mediating models were examined using SPSS PROCESS macro 3.3 software, in which the mediation variables were self-control, rumination, and psychological distress, and the moderation was loneliness. Gender, major, and grade were included as control variables.

**Result:**

Self-control, rumination, and psychological distress played a simple mediating role between physical exercise and MPA. Moreover, not only self-control and rumination but also self-control and psychological distress played the chain mediating roles between physical exercise and MPA. The chain pathways were moderated by loneliness. Specifically, the effect was more substantial among college students with higher loneliness.

**Conclusion:**

The conclusions corroborate and clarify that self-control, rumination, and psychological distress mediated the association between physical exercise and MPA, and the mediation effects were moderated *via* loneliness. This present study advanced our understanding of how and when college students’ physical exercise was related to MPA. It also illustrates that educators and parents should pay more attention to college students’ physical exercise.

## Introduction

Mobile phone user groups are growing rapidly with the development and popularity of mobile internet devices. Globally, 90% of people own mobile phones (Lian et al., 2021). According to the 49th statistical report released by the China Internet Network Information Center, as of December 2021, mobile phone users had reached 10.29 billion, and 99.7% of netizens used mobile phones to surf the Internet (Center, 2022). The mobile phone has become an integral part of human life. Particularly during the COVID-19 pandemic, due to the pandemic and the government’s policy of staying safe, people’s behavioral patterns and mental health were changed, and college students’ sedentary time and mobile phone use increased rapidly (Huckins et al., 2020). Among them, mobile phone use increased by 27.6% for men and 57.2% for women (Saadeh et al., 2021). It is reported that the mean prevalence of MPA in Chinese college students was 32%, indicating that excessive use of mobile phones by college students would increase the risk of MPA (Guo et al., 2022). As is known to all, MPA harms college students’ physical and mental health (Liu et al., 2022). Studies have found that MPA was closely linked to Physical pain (Demir and Sumer, 2019; Mustafaoglu et al., 2021), blurred vision (Liu et al., 2022), sleep quality (Demir and Sumer, 2019), depression, anxiety, and stress (Gao et al., 2018). In addition, MPA would seriously affect college students’ academic performance (Liu et al., 2022), interpersonal competence (Lee et al., 2018), and life quality (Liu et al., 2022). For instance, it is found that mobile phone users may experience high anxiety levels and poor academic performance (Andrew et al., 2014). Thereby, MPA has become a serious public health concern, and effective interventions are urgently needed to prevent MPA among college students.

MPA is also known as “unreasonable use of mobile phones” or “mobile phone dependence” (Liu et al., 2022), which is defined as uncontrollable use of mobile phones (Daniel, 2014), and similar non-substance addiction symptoms in mobile phone use were observed (Panova and Carbonell, 2018). Physical exercise, an essential part of a healthy life, is also a critical factor in preventing and managing mental illness and behavioral addictions (Marconcin et al., 2022). In recent years, physical activity has been proven to be an essential protective factor in MPA (Kim et al., 2015; Liu S. et al., 2019). For instance, a study of 1,433 college students in China showed that physical exercise negatively predicted MPA (Guo et al., 2022). When physical exercise increased from sedentary to moderate, the dose- dependent relationship between physical exercise and MPA was most apparent (Lian et al., 2021). In a 12-week intervention experiment involving Baduanjin and basketball, researchers found that physical exercise effectively reduced MPA among college students (Daniel, 2014). Moreover, among the psychological theories, the “distraction” argues that diversion from unpleasant stimuli or painful somatic complaint leads to improved emotion following exercise sessions (Morgan, 1985). The well-known mastery hypothesis and the self-efficacy theory focus on the post-exercise sense of revitalization and achievement, promoting positive moods (Marcus, 1995; Paluska and Schwenk, 2000). While in the Interaction of Person-Affect-Cognition-Execution (I-PACE) model, emotional and cognitive responses were the core characteristics of addictive behaviors (Brand et al., 2019). Therefore, individuals with moderate exercise would not devote extensive energy to problematic cell phone use (Tao et al., 2020). Previous studies have shown that physical exercise can reduce the negative emotions related to MPA (such as anxiety, depression, and stress; Grasdalsmoen et al., 2020) and has a positive role in the treatment of some psychological diseases and the suppression of MPA (Fan et al., 2021). Therefore, it is reasonable to believe that physical exercise is an essential protection against MPA.

In recent years, the negative correlation between physical exercise and MPA has been confirmed, but the link between mediating (i.e., how physical exercise relates to college students’ MPA) and moderating mechanisms (i.e., when physical exercise is the most effective intervention on MPA) needs further exploration. Based on the theory of the Interaction of Person-Affect-Cognition-Execution (I-PACE) model (Brand et al., 2019), time self-regulation of physical activity (Hall and Fong, 2015), Salmon’s unifying theory (Salmon, 2001) and compensatory Internet Use theory (Li J. et al., 2021), this study takes self-control, ruminant thinking, psychological distress, and loneliness as mediating and regulatory variables to construct a moderating mediation model.

### The mediating role of self-control

Self-control has been conceptualized as a state and a trait, which is a relatively stable trait associated with various positive outcomes (Tangney et al., 2004). Thus, this study focuses on the trait of self-control: “the stable ability to handle self-control dilemmas in such a way that the desired goal is prioritized” (Schuler et al., 2019). Under different conditions, due to overlapping definitions and structures, self-control has also been referred to as effort control, inhibitory control, cognitive control, and executive function (Duckworth et al., 2019). It can help individuals to quickly adjust themselves appropriately to adapt to the surrounding environment (Romero-Tena et al., 2021). Low self-control was closely related to drug abuse, addictive behavior, and maladjustment (Losada-Baltar et al., 2021). Empirical studies have proved that people with low self-control were more likely to develop MPA than those with high self-control ability (Xiang et al., 2019). When they lack self-control, individuals show more impulsive and irrational decisions (Testa et al., 2020). According to the addictive behavior model (I-PACE), the reduction of individual executive control and inhibitory control led to the reduction of motivation seeking and desire suppression, which led to excessive addictive behavior (Brand et al., 2016). The reduced inhibitory control was a vulnerability factor for addictive behaviors and a moderator of the relationship between specific emotional responses that trigger stimuli and the decision to engage in specific behaviors (Brand et al., 2019). Previous studies have also shown a positive correlation between low self-control and MPA (Jiang and Zhao, 2016). Therefore, lack of self-control was a significant risk factor for MPA.

Meanwhile, research on exercise psychology showed that physical exercise was one of the effective means to improve self-control (Guiney and Machado, 2013; Fan et al., 2021). Physical activity is closely related to executive function. According to the temporal self-regulation theory for physical activity, individuals with solid executive control are better able to engage in physical activity, which in turn helps to strengthen the executive control network (Hall and Fong, 2015). Some researchers regard MPA as a response to losing control over one’s body (Jiang and Zhao, 2016). Salmon’s unifying theory suggests that physical activity may enhance the executive functions controlling behavior, thoughts, and emotions (Salmon, 2001). The strength model of self-control suggests that self-control could be effectively improved or enhanced *via* regular physical activity or exercise (Yang et al., 2019). Previous studies have shown that different types and intensities of exercise positively affect self-control (Contreras-Osorio et al., 2021; Tian et al., 2021). For instance, one longitudinal study found that both acute and chronic physical exercise can be beneficial to the enhancement of self-control (Benzing et al., 2018). It is proved that high-intensity intermittent exercise could be a time-efficient approach for enhancing inhibitory control (Tian et al., 2021). Moreover, exercise might positively affect the cognitive control system of the brain (Smith et al., 2010; Dwyer et al., 2014). Physiological indicators showed that the cognitive control system was closely associated with the executive control network (ECN), commonly involved in executive control, working memory, and decision-making (Yin et al., 2022). Functional magnetic resonance imaging (fMRI) research studies have found that small metabolic changes in brain regions might be associated with executive function during physical exercise (Davis et al., 2011). Therefore, physical exercise may indirectly influence MPA through self-control (Hypothesis 1).

### The mediating role of rumination

Rumination refers to a mode of responding to distress that involves a repetitive focus on one’s distress, as well as its causes and consequences, rather than actively solving problems to relieve the negative emotions (Shaw et al., 2019). According to the response styles theory, rumination maintains and exacerbates negative moods by enhancing negative thinking (Shaw et al., 2019). Numerous studies suggest that rumination was an influential factor in depressive symptoms, anxiety (Candea and Szentagotai-Tatar, 2017), perceived stress (Ruscio et al., 2015), and suicidal ideation (Ruscio et al., 2015; Candea and Szentagotai-Tatar, 2017; Holdaway et al., 2018). Given that depression, anxiety, and stress are important risk factors for MPA (Elhai et al., 2020a,b; Gao et al., 2018), rumination may induce and exacerbate MPA. Studies on the relationship between rumination and MPA have also confirmed that rumination was an important predictor of MPA (Peng et al., 2022). For instance, in a cross-sectional study of rumination and MPA, rumination can further predict MPA with the enhancement of excessive assurance-seeking behavior (Elhai et al., 2020a,b).

Given that rumination is a risk factor for MPA (Elhai et al., 2020a,b), various factors that may prevent rumination have been of great concern. Physical exercise has been shown to be effective as a monotherapy for rumination (Cooney et al., 2013) and a psychotherapy enhancement strategy (Abdollahi et al., 2017). According to Salmon’s unified theory, physical exercise can improve cognitive responses, arouse benign attributions of fear stimuli, and prevent panic factors caused by negative emotions (Salmon, 2001). Previous studies have shown that exercise reduces rumination and affects depressive symptoms in general (Bernstein and Mcnally, 2017). In biology, studies have reported evidence for increased neuroplasticity, especially in the hippocampus, which is relevant to cognitive-emotional processing (Medina et al., 2015; Kandola et al., 2019). The research identified that physical exercise increases neuroplasticity in the hippocampal circuit, and changes in this circuit may allow individuals to process emotional information differently to reduce the automatic prioritization of negative information and thus reduce rumination (Hamilton and Gotlib, 2008). In addition, studies have found that rumination in patients with mental disorders is negatively associated with aerobic exercise (Brand et al., 2018). Physical exercise plays a positive role in the improvement of rumination. Therefore, rumination could be regarded as a mediator between physical exercise with MPA (Hypothesis 2).

### The mediating role of psychological distress

Psychological distress is a state of emotional distress associated with anxiety, depression, stress, and general mood disorders, reflecting the internal state of an individual’s mental health (Wong et al., 2014; Liu R.D. et al., 2019). The cause of psychological distress may be related to individual needs that are not met (Wong et al., 2014). According to compensatory Internet use theory, psychologically troubled individuals tend to use the Internet to deal with negative emotions or compensate for problems in reality (Daniel, 2014). For instance, due to social difficulties and negative emotions caused by shyness, individuals may resort to the online world to relieve negative emotions and meet their needs (Cole et al., 2019). Once relief and satisfaction can be obtained from mobile phone interaction, people are more likely to view mobile phone use as a useful coping strategy, leading to automatic activation and potentially addictive behavior (Nicholls et al., 2014). Cognitive behavioral models show that psychological distress such as depression, anxiety, and other negative emotions are risk factors for problematic Internet use (Davis, 2001). A growing number of studies have also confirmed a positive correlation between psychological distress and MPA (Liu R.D. et al., 2019). For example, a cross-sectional study found that psychological distress, such as anxiety and depression, can predict problematic internet use (Arrivillaga et al., 2022), which might increase the risk of MPA (Geng et al., 2021). In addition, with the increase in stress, depression, and anxiety, the level of MPA was also increased (Gao et al., 2018). Therefore, it is reasonable to postulate that psychological distress is closely associated with increased MPA.

It is well known that physical exercise improves physical and mental health. Physical exercise, as a non-drug intervention for depression and anxiety symptoms, has attracted researchers` attention (Brondino et al., 2017). For example, a meta-analysis reported a significant effect of physical exercise in alleviating depressive symptoms (Kvam et al., 2016). At the same time, some studies have found that depression, anxiety, and other negative emotions are closely related to cognitive dysfunction (Shilyansky et al., 2016), and that physical exercise could positively affect cognitive function. For instance, in terms of biology, it has been found that physical exercise can promote prominent plasticity in the hippocampus through brain-derived neurotrophic factor (BDNF), and elevate BDNF levels, thus promoting cognitive development (Aguiar et al., 2011). Empirical studies have shown that physical exercise was associated with cognitive improvement in individuals with mild cognitive impairment (Zheng et al., 2016). Therefore, physical exercise can relieve psychological distress by improving cognition. Meanwhile, according to salmon’s unified theory, physical exercise may trigger more beneficial processes that indirectly improve mental health (Salmon, 2001), such as enhancing the executive ability to control behavior (Wang et al., 2022), thoughts, emotions, and enhancing resistance to physical and emotional consequences of psychological stressors (Audiffren and Andre, 2019). In the Boehm and Kubzansky model, exercise is classified as a restorative behavior associated with psychological distress (Boehm and Kubzansky, 2012). A population-based longitudinal study also suggests that light and moderate physical activity can protect against future psychological distress (Sheikh et al., 2018). Therefore, this study hypothesized that psychological distress might act as an intermediary linking physical exercise and MPA (Hypothesis 3).

### The chain mediating roles of self-control and rumination

Numerous studies have revealed the protective effects of attention control, cognitive control, executive function, and mindfulness on rumination (DeJong et al., 2019; du Pont et al., 2019). Although few studies have shown a direct relationship between self-control and rumination, self-control has also been justified as a predictor of rumination (Breithaupt et al., 2016). According to the response style theory of rumination, rumination is closely associated with the cognitive ability to promote goal-related behaviors by regulating thoughts and behaviors (Friedman and Miyake, 2017). The impaired disengagement hypothesis suggests that low levels of attentional control led to prolonged and habitual rumination (du Pont et al., 2019). For instance, most people experience negative and critical self-focused thoughts as incongruent with their positive self-image, which leads to conflicting signals from the negative thoughts (Koster et al., 2011). However, disruptions of conflict signaling processes (e.g., with reduced attentional control) can lead to a sustained focus on negative thoughts and habitual engagement in rumination (De Raedt and Koster, 2010). The dual process model of cognitive land vulnerability and resource allocation hypothesis suggests that rumination and limited cognitive resources require cognitive control (Levens et al., 2009). These hypotheses all reflect the correlation between self-control and rumination. Meanwhile, studies have found that aerobic exercise combined with meditation significantly enhances cognitive control processes and reduces rumination patterns (Lavadera et al., 2020). Based on these accumulated findings, physical exercise may indirectly influence MPA through the chain mediating effect of self-control and rumination (Hypothesis 4).

### The chain mediating roles of self-control and psychological distress

Given that psychological distress is a risk factor for MPA and self-control is associated with mental health (Cheung et al., 2014; Gao et al., 2018), this study further explored the protective effect of self-control on psychological distress. Self-control is always defined as the capacity to alter the predominant response to promote desirable long-term goals (Li et al., 2015), which was associated with many mental health indicators such as satisfaction with life, happiness and self-esteem (Cheung et al., 2014). Which has also been linked to lower levels of depression and anxiety (Li et al., 2019; Liu R.D. et al., 2019). Previous studies have found that high levels of self-control can protect teenagers from psychological distress. Cognitive theories of emotion suggest that self-control plays a vital role in adaptive and maladaptive emotional processes (Ainsworth and Garner, 2013). This is supported by empirical studies indicating that higher anxiety was associated with inhibitory control deficits in individuals (Ansari and Derakshan, 2011). Furthermore, a meta-analysis has shown that lower self-control is associated with increased depression and anxiety (Ran et al., 2019). Therefore, self-control plays a positive role in reducing psychological distress. Based on the above research results, we hypothesize that physical exercise may indirectly affect MPA through the chain mediating effect of self-control and psychological distress (Hypothesis 5).

### The moderating role of loneliness

Although physical exercise might be related to MPA through self-control and rumination or self-control and psychological distress, this effect may vary according to individual characteristics. According to the Interaction of Person-Affect-Cognition-Execution (I-PACE) model, a person’s characteristics may influence their internal responses (execution, cognitive response, and affect), leading to the establishment and intensification of problematic behavioral outcomes (Li J. et al., 2021). Loneliness as a personality trait has attracted more and more attention (Li X. et al., 2021). Thus, we would further introduce loneliness as individual factor and investigate whether the relationship between physical exercise and MPA can be buffered by loneliness.

Loneliness is an experience of negative emotional experience caused by an interpersonal relationship gap and the resulting emotions (Fan et al., 2022). As described by multidimensional models of loneliness, this negative experience has far-reaching affective (e.g., depression), cognitive (e.g., maladaptive perceptions), and behavioral consequences (e.g., risk-related behavior), which negatively impact psychological and physiological health and well-being (Arpin and Mohr, 2019). Firstly, loneliness may moderate the positive effect of self-control on rumination. Loneliness is related to the ability of individuals to process emotions and regulate their feelings, and high levels of loneliness may prolong or deepen the negative emotions felt and increase the risk of rumination (Hards et al., 2022). According to the ruminative stress response model, individuals who experience more negative experiences (e.g., Loneliness,) may ruminate more about their life and emotional states (Tong et al., 2021). Loneliness has been shown to be a crucial risk factor in rumination (Tong et al., 2021). In a cross-sectional study of young adults, high loneliness is usually correlated with low levels of positive emotion and other features that reflect high levels of negative emotion (Zawadzki et al., 2013). A longitudinal study of older adults found that individuals with high levels of loneliness tend to experience more rumination (Gan et al., 2015). Therefore, at the same level of self-control, individuals with high loneliness will acquire more rumination, and the effect of self-control ability on rumination will be enhanced. That is, as loneliness increases, the relationship between self-control and rumination becomes stronger. Therefore, loneliness might act as a buffer in the link between self-control and rumination (Hypothesis 6a).

Secondly, Loneliness may also buffer the influence of self-control on psychological distress. A substantial body of empirical studies has confirmed that loneliness reduces the ability of individual emotion regulation. For instance, a systematic review found that loneliness is associated with worse mental health outcomes, including worse depression and anxiety symptoms and poorer remission of depression (Hards et al., 2022). Many studies have shown that loneliness was a significant predictor of psychological distress, with people who report more loneliness also reporting more depression and higher stress levels (Lam et al., 2017; Yung et al., 2021). This finding was supported by studies of older adults and adolescents. Longitudinal studies of loneliness in older adults predicted depressive symptoms 2–12 years later (Lee et al., 2020). A similar phenomenon has been found in studies of young adults and adolescents (Qualter et al., 2010). A meta-analysis of 63 studies found that loneliness had a negative impact on the mental health of adolescents and young adults for up to 9 years, with the most significance on depression (Loades et al., 2020). Many longitudinal studies have found a correlation between loneliness and depression, and the relationship was more robust in the early stages of depression (Hsueh et al., 2019; Santini et al., 2020). Therefore, increased loneliness raises the risk of psychological distress. In addition, loneliness is accompanied by a social environment of isolation and loss of support. Individuals without social support are in a fragile psychological state, leading to the onset or amplification of psychological distress (Hards et al., 2022). According to the interpersonal theory and stress generation theory, increasing social isolation and loneliness will increase the individual’s response to stressors, anxiety, depression, and other negative emotions (Flynn et al., 2010). Therefore, low social support and high levels of loneliness enhance the effects of high self-control on psychological distress. As loneliness increased, the relationship between self-control and psychological distress became more pronounced. Therefore, self-control might be able to moderate the relationship between self-control and psychological distress (Hypothesis 6b).

### The present study

**Figure 1:**
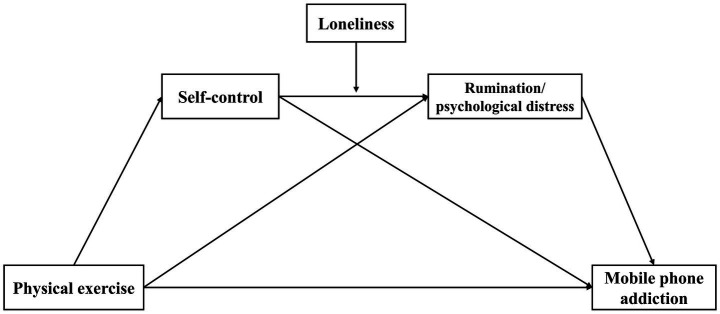
The proposed model.

Considering the harm of MPA to college students’ physical and mental health, it is imperative to examine the protective mechanism of physical exercise on MPA. Thus, the present study examined the direct effects of physical exercise on MPA in college students and assessed whether any detected association of physical exercise with MPA was mediated by self-control, rumination, and psychological distress and modulated by loneliness. The proposed model is illustrated in Figure 1. The specific assumptions were as follows:

*Hypothesis 1:* Self-control plays a mediating role between Physical Exercise and MPA. (H1)

*Hypothesis 2:* Rumination plays a mediating role between Physical Exercise and MPA. (H2)

*Hypothesis 3:* Psychological Distress plays a mediating role between Physical Exercise and MPA. (H3)

*Hypothesis 4:* Self-control and Rumination play a chain mediating role between Physical Exercise and MPA. (H4)

*Hypothesis 5:* Self-control and Psychological Distress play a chain mediating role between Physical Exercise and MPA. (H5)

*Hypothesis 6a:* Loneliness modulates the chain mediating role of Self-control and Rumination. (H6a)

*Hypothesis 6b:* Loneliness modulates the chain mediating role of Self-control and Psychological Distress. (H6b)

## Materials and methods

### Participants and procedures

**Figure 2:**
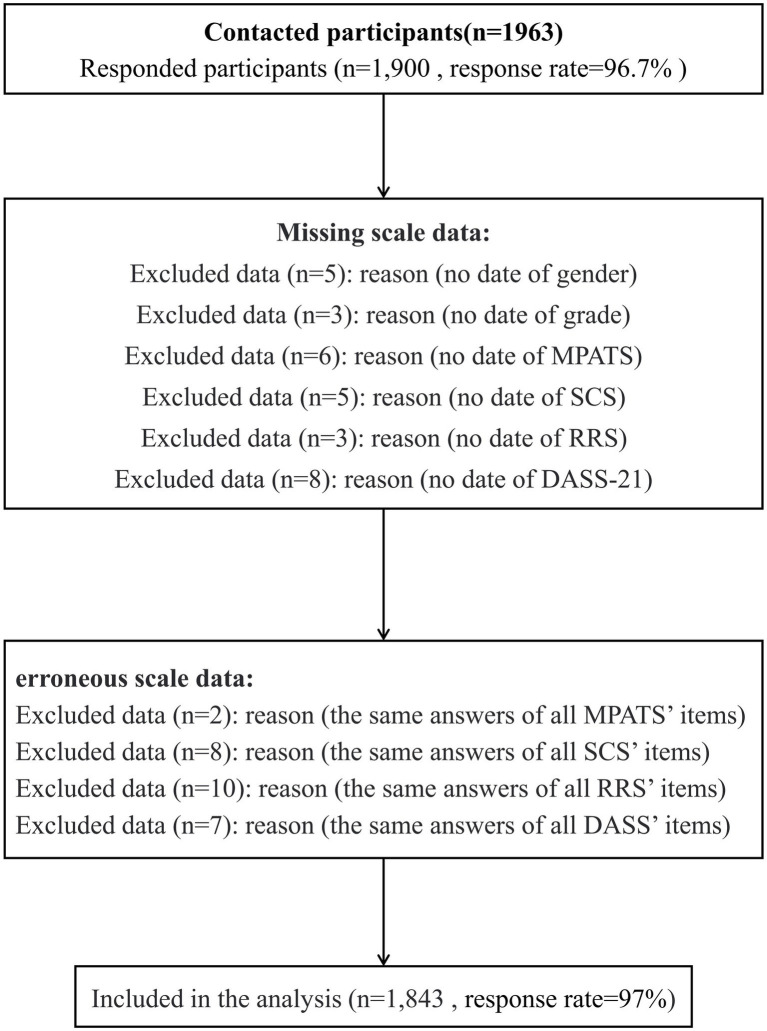
The procedure of obtaining final study sample.

A cross-sectional survey was conducted by using the convenience sampling method, in five universities in Sichuan province from October to November 2021. Participants were recruited before class and asked to complete paper questionnaires in class. We recruited 1,963 college students aged 17–27 years. More details of the selection process are outlined in Figure 2. The response sample (*n* = 1,900) included a total of 1,843 participants (56.9% female) in the final analysis. The effective response rate was 97%. The mean age of the participants was 19.75 years (SD = 1.3). Participants understood the requirements of the survey through personal explanation, and all questionnaires were completed within 30 min. The study followed the guidelines of the STROBE checklist, complied with the principles of the Declaration of Helsinki, and it is supported and approved by the Institutional Review Board of Sichuan University. Signed informed consent forms were obtained from students.

### Measures

#### Mobile phone addiction

The Mobile Phone Addiction Tendency Scale (MPATS) was applied to estimate the MPA for college students (Jie et al., 2012), which has been used in Chinese college students and young adults with good reliability and validity (Yang et al., 2019). The MPATS is a 5-point-Likert scale consisting of 16 items and four dimensions: withdrawal symptoms (WS), salience behavior (SB), social comfort (SC), and mood changes (MC; e.g., “I would rather chat on my cell phone than face to face”). Each item is rated from 1 (completely disagree) to 5 (completely agree), and the total score will be from 16 to 80, and a higher score may mean a deeper degree of MPA (Kimberly, 1998). The internal consistency coefficient and retest reliability of MPATS were 0.83 and 0.91, respectively, (Jie et al., 2012). In the present study, confirmatory factor analysis results demonstrated that a single-factors model fit the data satisfactorily: *χ*^2^/df = 5.29, CFI = 0.97, TLI = 0.95, RMSEA = 0.048, SRMR = 0.038, and the Cronbach’s *α* was 0.88.

#### Physical exercise

Physical exercise (PE) was measured by the Physical Activity Rating Scale-3 (PARS-3; Yang et al., 2021), which has been used in Chinese college students and young adults with good reliability and validity (Yang et al., 2021). The PARS-3 is a three-item self-reported scale containing exercise intensity, exercise time, and exercise frequency (e.g., “How hard do you exercise?”; Yang et al., 2019). Each item is rated from 1 to 5, and the following equation computes the total score of physical activity: intensity × (time-1) × frequency, with a range of 0–100. The PARS-3 has excellent test–retest reliability (*r* = 0.82; Yang et al., 2019). The internal consistency of PARS-3 in this study was basically satisfactory, and the Cronbach’s *α* was 0.6.

#### Self-control

Self-control (SC) was evaluated by the self-control scale (SCS; Yang et al., 2019), which was modified based on Tangney’s Self-Control Scale and showed good reliability and validity among Chinese college students (Tangney et al., 2004; Geng et al., 2021). The SCS is a 5-point-Likert scale and comprises 19 items. It has five dimensions: controlling impulses, keeping healthy habits, resisting temptation, concentrating on work, and controlling entertainment. Fifteen items were scored in reverse, and four were scored in a positive direction (e.g., “It is difficult for me to break bad habits”). Each item is valued from 1 (completely disagree) to 5 (completely agree). The total score can be from 19 to 95, and a higher score shows a higher level of individual self-control (Ma et al., 2020). The SCS has a fair internal consistency coefficient (*α* = 0.86) and retest reliability (*r* = 0.89; Jiang and Zhao, 2016). In the present study, confirmatory factor analysis results demonstrated that a single-factors model fit the data satisfactorily: *χ*^2^/df = 5.01, CFI = 0.96, TLI = 0.93, RMSEA = 0.047, SRMR = 0.04, and the Cronbach’s *α* was 0.85.

#### Rumination

Rumination (RA) was measured by the Chinese version of the Ruminative Response Scale (RRS; Lei et al., 2017) developed by Nolen-Hoeksema (1991) and showed good reliability and validity among Chinese college students (Lian et al., 2021). Participants responded to the 22 items on a Likert-type scale ranging from 1 (never) to 4 (always; e.g., “Go some-place alone to think about your feelings” and “I often think about the situation and wish it would change for the better”). Higher scores reflect a higher tendency to respond to negative factors with a ruminative response style (Lei et al., 2017). In this study, confirmatory factor analysis results demonstrated that a single-factors model fit the data satisfactorily: *χ*^2^/df = 5.21, CFI = 0.97, TLI = 0.95, RMSEA = 0.048, SRMR = 0.02, and the Cronbach’s *α* was 0.931.

#### Psychological distress

Psychological distress (PD) was assessed by the Chinese version of Depression Anxiety Stress Scale-21 (DASS-21), which has been widely used to measure individual psychological distress (Wang et al., 2016) and showed good reliability and validity in Chinese samples (Li Y. et al., 2021). The scale consists of 21 items that cover three subscales: depressive symptoms scale (e.g., “I could not seem to experience any positive feeling at all”), anxiety symptoms scale (e.g., “I was aware of dryness of my mouth”), and stress symptoms scale (e.g., “I found it hard to wind down”). All items were rated on a four-point scale ranging from 0 (did not apply to me at all) to 3 (applied to me very much or most of the time). The higher the score, the more psychological distress (Chen et al., 2021). In this study, confirmatory factor analysis results demonstrated that a single-factors model fit the data satisfactorily: *χ*^2^/df = 5.50, CFI = 0.96, TLI = 0.95, RMSEA = 0.049, SRMR = 0.02, and the Cronbach’s *α* was 0.933.

#### Loneliness

Loneliness was assessed using the Chinese version of the UCLA Loneliness Scale (Jianfeng et al., 2016), which was modified based on Russell’s UCLA Loneliness Scale (Russell, 1996) and showed good reliability and validity among Chinese college students (Xia and Yang, 2019). It consists of a total loneliness scale and three subscales that correspond to three self-related facets of loneliness and social connectedness: Isolation, Relational Connectedness, and Collective Connectedness. Participants responded to 20 questions on a Likert-type scale (e.g., Are you lonely?; Padmanabhanunni and Pretorius, 2021). All items are rated on a four-point scale, from 1 (never) to 4 (always). Furthermore, 11 items in the project are scored forward, and nine items are scored backward; the higher the score, the more loneliness. In the present study, confirmatory factor analysis results demonstrated that a single-factors model fit the data satisfactorily: *χ*^2^/df = 5.94, CFI = 0.95, TLI = 0.92, RMSEA = 0.052, SRMR = 0.038, and the Cronbach’s *α* was 0.88.

### Statistical analyses

Amos 24.0 was used for confirmatory factor analysis to test the validity of variables, and SPSS 26 was used to study descriptive statistics, Pearson correlation, and bias analysis of common methods. Descriptive characteristics of the participants are presented as means (M) and standard deviation (SD). Gender, major, and grade were selected as covariates since they were associated with the main variables. Partial correlation coefficients were estimated to examine the associations among physical exercise, self-control, rumination, psychological distress, loneliness, and MPA. Harman’s single-factor test was used to test for common method bias. According to the study’s recommendation (Qian et al., 2022). Using process macros in SPSS26 to examine our model. Firstly, we used process model 4 to test the simple mediating models of self-control, rumination, and psychological distress. Secondly, using model 6 to test the chain mediated model of self-control, rumination, and psychological distress. Next, model 91 is applicable to test the moderated mediation effect in the conceptual model shown in Figure 1. Mediation and moderation hypotheses were tested with bootstrapping using resampling of 5,000 samples to calculate 95% confidence intervals (CIs). The results were deemed statistically significant if the 95% CI did not contain zero and the *p* value was <0.05. In addition, this study referred to effect sizes of the correlation coefficient *r* (Yang et al., 2021) to estimate the magnitude of significant differences during statistical analysis.

## Results

### Common method deviation test

Because the data in this study was obtained in the form of questionnaire self-report, to avoid common method bias, Harman’s single-factor test was used to test the bias of common methods (Gao et al., 2020; Peng et al., 2020). The results show that the original roots of 17 factors are more significant than 1. The cumulative variance explained by the first factor was 9.44% (the threshold was 40%). This indicates that this study has no major problems with common methodological bias.

### Confirmatory factor analysis

Before testing the hypothesis, we used confirmatory factor analysis (CFA) to validate the measurement model (Li and Peng, 2022). The measurement model includes five potential factors: physical exercise, self-control, rumination, psychological distress, and MPA. The CFA results of this study are shown in Table 1. Results showed that the data of the five-factor model were in good fit [*χ*^2^ (87) = 788.384, values of CFI = 0.945, TLI = 0.924, SRMR = 0.03, RMSEA = 0.066]. This proved that the model’s goodness of fit is significantly better than other factor models. These results of CFAs provided full support for the discriminate validity of our study instruments.

**Table 1:** Results of confirmatory factor analyses.

Models	Variables	*χ* ^2^	df	*c*^2^/df	CFI	TLI	SRMR	RMSEA
Five-factor model	PE, SC, RA, PD, MPA	788,384	87	9.062	0.945	0.924	0.03	0.066
Four-factor model	PE, SC, RA + PD, MPA	1620.45	98	16.535	0.88	0.853	0.32	0.092
Three-factor model	PE, SC, RA + PD + MPA	2807.595	101	27.798	0.787	0.747	0.054	0.121
Two-factor model	PE + SC, RA + PD + MPA	3452.441	103	33.519	0.736	0.693	0.087	0.133
Single-factor model	PE + SC + RA + PD + MPA	4488.229	104	43.156	0.655	0.601	0.092	0.151

PE, physical exercise; MPA, mobile phone addiction; SC, self-control; RA, Rumination; PD, psychological distress.

### Primary analysis

Table 2 shows all observed variables’ mean, standard deviation, and correlation. Physical exercise was positively correlated with self-control and negatively correlated with MPA, rumination, and psychological distress. Self-control was negatively correlated with MPA, rumination, and psychological distress. Loneliness was positively correlated with rumination and psychological distress. Rumination and psychological distress were positively correlated with MPA.

**Table 2:** Descriptive statistics and interrelations among of the observed variable.

**Variable**	**M**	**SD**	**1**	**2**	**3**	**4**	**5**	**6**	**7**	**8**	**9**
1. Gender	1.57	0.50	1								
2. Major	1.98	1.48	−0.20^**^	1							
3. Grade	1.60	0.64	0.10^**^	0.03	1						
4. PE	27.08	23.49	−0.29^**^	0.51^**^	−0.04	1					
5. MPA	2.61	0.66	0.18^**^	−0.06^*^	0.09^**^	−0.14^**^	1				
6. SC	3.12	0.54	−0.17^**^	0.09^**^	−0.09^**^	0.15^**^	−0.45^**^	1			
7. RA	2.09	0.53	0.03	−0.04	0.04	−0.08^**^	0.36^**^	−0.41^**^	1		
8. PD	1.72	0.53	−0.03	0.01	0.03	−0.05^*^	0.37^**^	−0.45^**^	0.70^**^	1	
9. LN	2.16	0.43	0.06^*^	−0.02	−0.001	−0.12^**^	0.26^**^	−0.27^**^	0.46^**^	0.49^**^	1

*N* = 1,843. PE, physical exercise; MPA, mobile phone addiction; SC, self-control; RA, Rumination; PD, psychological distress; LN, loneliness.

** *p* < 0.01;

* *p* < 0.05.

### Testing for the simple mediation model

The results are shown in Table 3. As expected, the overall effect of physical exercise on MPA was significant in all simple mediation models (*β* = −0.11, *p* < 0.001). In the mediating model of self-control, physical exercise has a positive predictive effect on self-control (*β* = 0.094, *p* < 0.001), self-control (*β* = −0.43, *p* < 0.001), and physical exercise (*β* = −0.066, *p* < 0.01) negatively predicts MPA. In the mediating model of rumination, physical exercise has a negative predictive effect on rumination (*β* = −0.072, *p* < 0.01), rumination (*β* = 0.35, *p* < 0.001) positively predicts MPA, and physical exercise (*β* = −0.08, *p* < 0.01) negatively predicted MPA. In the mediating model of psychological distress, physical exercise has a negative predictive effect on psychological distress (*β* = −0.084, *p* < 0.01), Psychological distress (*β* = 0.37, *p* < 0.001) positively predicts MPA, and physical exercise (*β* = −0.08, *p* < 0.01) negatively predicted MPA. The bias-corrected bootstrapping mediation test indicated the process of physical exercise predicting MPA through self-control, rumination, and psychological distress were significant in the simple mediation model. Therefore, hypothesis 1, hypothesis 2, and hypothesis 3 were supported.

**Table 3:** Regression analysis of the simple mediation model.

Variable	Outcome: SC			Outcome: RA			Outcome: PD			Outcome: MPA											
										Total			Mediator: SC			Mediator: RA			Mediator: PD		
	*B*	SE	*p*	*B*	SE	*p*	*B*	SE	*p*	*B*	SE	*p*	*B*	SE	*p*	*B*	SE	*p*	*B*	SE	*p*
1 Gender	−0.13^**^	0.05	*p* < 0.001	0.01	0.05	0.8	−0.05	0.05	0.05	0.15^***^	0.05	*p* < 0.001	0.1^***^	0.04	*p* < 0.001	0.15^***^	0.05	*p* < 0.001	0.17^***^	0.045	*p* < 0.001
2 Major	0.02	0.02	0.38	−0.01	0.02	0.89	0.04	0.02	0.12	0.03	0.02	0.33	0.04	0.02	0.14	0.03	0.02	0.28	0.01	0.02	0.68
3 Grade	−0.07^**^	0.04	*p* < 0.01	0.04	0.04	0.12	0.03	0.04	0.25	0.07^**^	0.04	*p* < 0.01	0.042^*^	0.03	*p* < 0.05	0.06^**^	0.03	*p* < 0.01	0.06^**^	0.03	*p* < 0.01
4 PE	0.09^***^	0.03	*p* < 0.001	−0.07^**^	0.03	*p* < 0.01	−0.08^**^	0.03	*p* < 0.01	−0.11^***^	0.03	*p* < 0.001	−0.07^**^	0.02	*p* < 0.01	−0.08^**^	0.03	*p* < 0.01	−0.08^**^	0.03	*p* < 0.01
5 SC													−0.43^***^	0.02	*p* < 0.001	–	–	–	–	–	–
6 RA													–	–	–	0.35^***^	0.02	*p* < 0.001	–	–	–
7 PD													–	–	–	–	–	–	0.4^***^	0.02	*p* < 0.001
**Results of bootstrapping mediation effect examination**																					
**Mediating effects**					**indirect**		**95%CI**						**Proportion**			**Degree of mediation**					
PE → SC → MPA (H1)					−0.04^**^		[−0.065, −0.017]						37.73%			Partial mediation					
PE → RA → MPA (H2)					−0.025^**^		[−0.046, −0.006]						23.36%			Partial mediation					
PE → PD → MPA (H3)					−0.031^**^		[−0.052, −0.011]						28.97%			Partial mediation					

*N* = 1,843. M, meditation; PE, physical exercise; MPA, mobile phone addiction; SC, self-control; RA, Rumination; PD, psychological distress. Unstandardized regression coefficients are reported. Bootstrap sample size = 5,000.

*** *p* < 0.001;

** *p* < 0.01;

* *p* < 0.05.

### Testing for the chain mediation model

The path statistics are presented in Table 4. The overall effect of physical exercise on MPA was significant in all chain mediation models (*β* = −0.11, *p* < 0.001). In the serial mediating effecting, physical exercise can positively predict self-control (*β* = −0.094, *p* < 0.001), self-control negatively predicted rumination (*β* = −0.41, *p* < 0.001) and psychological distress (*β* = −0.46, *p* < 0.001), rumination positively predicted MPA (*β* = 0.22, *p* < 0.001), psychological distress positively predicted MPA (*β* = 0.23, *p* < 0.001). The bias-corrected bootstrapping mediation test indicated that it was significant, the process of physical exercise predicting MPA through self-control and rumination (ab = −0.008, Boot SE = 0.003, 95% CI = [−0.014, −0.003]), and through the self-control and psychological distress (ab = −0.01, Boot SE = 0.003, 95% CI = [−0.017, −0.004]). Therefore, hypothesis 4 and hypothesis 5 were supported.

**Table 4:** Regression analysis of the chain mediating model.

variable	Outcome: SC			Outcome: RA			Outcome: PD			Outcome: MPA								
										Total			mediator: SC-RA			mediator: SC-PD		
	*B*	SE	*p*	*B*	SE	*p*	*B*	SE	*p*	*B*	SE	*p*	*B*	SE	*p*	*B*	SE	*p*
1 Gender	−0.13^***^	0.05	*p* < 0.001	−0.05^*^	0.05	*p* < 0.05	−0.11^***^	0.04	*p* < 0.001	0.15^***^	0.05	*p* < 0.001	0.11^***^	0.04	*p* < 0.001	0.12^***^	0.04	*p* < 0.001
2 Major	0.02	0.02	0.38	0.01	0.02	0.81	0.05^*^	0.02	*p* < 0.05	0.03	0.02	0.33	0.04	0.02	0.14	0.02	0.02	0.31
3 Grade	−0.07^**^	0.04	*p* < 0.01	0.01	0.03	0.72	−0.01	0.03	0.78	0.072^**^	0.04	*p* < 0.01	0.04^*^	0.03	*p* < 0.05	0.04^*^	0.03	*p* < 0.05
4 PE	0.09^***^	0.03	*p* < 0.001	−0.03	0.03	0.19	−0.04	0.02	0.1	−0.11^***^	0.03	*p* < 0.001	−0.06^*^	0.02	*p* < 0.05	−0.06^*^	0.02	*p* < 0.05
5 SC				−0.41^***^	0.02	*p* < 0.001	−0.46^***^	0.02	*p* < 0.001				−0.34^***^	0.02	*p* < 0.001	−0.32^***^	0.02	*p* < 0.001
6 RA													0.22^***^	0.02	*p* < 0.001	-	-	-
7 PD																0.23^***^	0.02	*p* < 0.001
*R* ^2^		0.043			0.168			0.21			0.047			0.26			0.26	
*F*		20.57^***^			73.93^***^			98.4^***^			22.77^***^			108.57^***^			109.39^***^	
**Results of bootstrapping mediation effect examination**																					
**Mediating effects**					**indirect**		**95%CI**						**Proportion**			**Degree of mediation**					
PE → SC → RA → MPA (H4)					−0.008^**^		[−0.014, −0.003]						17.40%			partial mediation					
PE → SC → PD → MPA (H5)					−0.01^**^		[−0.017, −0.004]						19.76%			partial mediation					

*N* = 1,843. M, meditation; PE, physical exercise; MPA, mobile phone addiction; SC, self-control; RA, Rumination; PD, psychological distress. Unstandardized regression coefficients are reported. Bootstrap sample size = 5,000.

*** *p* < 0.001;

** *p* < 0.01;

* *p* < 0.05.

### Testing for the moderated mediation model

**Figure 3:**
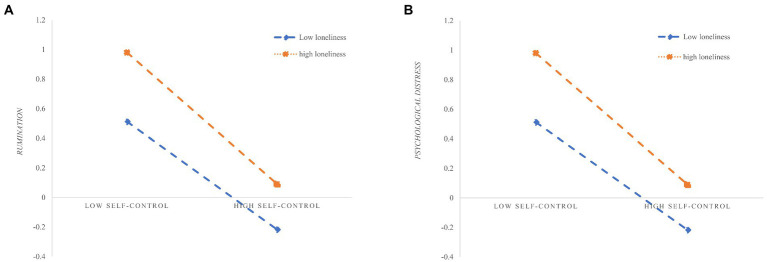
**(A)** Loneliness moderates the relation between self-control and rumination. **(B)** Loneliness moderates the relation between self-control and psychological distress.

Figure 3 shows the main results. As shown in Figures 3A,B, self-control x loneliness interaction had significant effects on rumination (*β* = −0.056, *p* < 0.001) and psychological distress (*β* = −0.081, *p* < 0.001). These findings indicated that both the association between self-control and rumination and between self-control and psychological distress were moderated by loneliness. In addition, simple slope analyses were conducted to illustrate these significant interactions and explore whether slopes for the high-loneliness group (1 SD above the mean) were different from slopes for the low-loneliness group (1 SD below the mean) in the two models. The results were plotted in Figures 3A,B. It showed the relationship between self-control and rumination as well as self-control and psychological distress at two loneliness levels (M + 1SD and M − 1SD). As shown in the figure, self-control was negatively correlated with rumination (*β* = −0.26, *t* = −10.0, *p* < 0.001) and psychological distress (*β* = −0.28, *t* = −11.6, *p* < 0.001) for college students with low loneliness (M − 1SD). In addition, for college students with high loneliness (M + 1SD), self-control was stronger negatively correlated with rumination (*β* = −0.37, *t* = −0.37, *p* < 0.001) and psychological distress (*β* = −0.45, *t* = −16.98, *p* < 0.001). In other words, regardless of the degree of self-control, students with higher levels of loneliness reported higher levels of rumination and psychological distress, while students with lower levels of loneliness reported lower levels of rumination and psychological distress. Therefore, hypothesis 6a and hypothesis 6b were supported (Table 5).

**Table 5:** Regression results of moderated mediation.

	*R* ^2^	*F*	df_1_	df_2_	*p*	*B*	Boot SE	*t*	*p*
SC * LN → RA	0.31	115.34	7	1,835	<0.001	−0.056^***^	0.02	−3.38	<0.001
SC * LN → PD	0.36	149.28	7	1,835	<0.001	−0.08^***^	0.02	−5.08	<0.001
The conditional effect analysis of loneliness value (M ± SD) between self-control and rumination									
						***B***	**Boot SE**	**Boot LLCL**	**Boot ULCL**
M – 1SD (−1)						−0.26	0.03	−0.31	−0.21
M (0)						−0.31	0.02	−0.35	−0.27
M + 1SD (1)						−0.37	0.03	−0.42	−0.31
The conditional effect analysis of loneliness value (M ± SD) between self-control and psychological distress									
						***B***	**Boot SE**	**Boot LLCL**	**Boot ULCL**
M – 1SD (−1)						−0.28	0.02	−0.33	−0.24
M (0)						−0.36	0.02	−0.4	−0.33
M + 1SD (1)						−0.45	0.03	−0.5	−0.39

*N* = 1,843. M, mean; SD, Standard deviation; PE, physical exercise; MPA, mobile phone addiction; SC, self-control; RA, Rumination; PD, psychological distress; LN, loneliness.

*** *p* < 0.001.

## Discussion

With the continuous progress of technology and the normalized development of the epidemic, mobile phones have become an indispensable part of daily life (Zhang and Wu, 2020). The problem of addiction caused by excessive use of mobile phones is also becoming more and more common among college students (Xiang et al., 2019). Recent studies have found that physical exercise positively protects MPA (Guo et al., 2022). However, more studies are needed to reveal the exact mechanisms. Based on theory and practice study, the study formulated the moderated mediation model to find the intrinsic relationship between physical exercise and MPA, and they also provide insights into the intervention of behaviors of MPA among undergraduates.

In the study, physical exercise is negatively associated with MPA and could positively predict MPA. The study reinforces the relationship between physical activity and MPA. Active participation may decrease screen time and sedentary behavior among college students so that they have less time to devote to mobile phone use and a lower chance of becoming addicted to it (Li Y. et al., 2021). According to the theory of Ternary Interaction, the environment, the individual and behavior influence each other (Flynn et al., 2010). As a significant external environmental stimulus, physical exercise can not only effectively improve individual physical health but also play an essential role in reducing behavioral addiction (Guo et al., 2022). In addition, the distraction hypothesis holds that the stimulation of physical exercise can divert the individual’s attention to negative emotions and then replace the effect of mobile phone use in diverting the individual’s negative emotions (Marconcin et al., 2022). From a neurophysiological perspective, studies have found that physical exercise can restore and adjust highly excited nerve cells, improving mobile phone addicts’ adaptability to external changes (Guo et al., 2022). In addition, physical exercise and cell phone use can activate similar neurobiological pathways in the brain. For example, inducing similar reward-based effects, activating brain regions associated with reward, and promoting dopamine release (Liu S. et al., 2019). Long-term exercise increases reward-related neural plasticity in brain structures, such as the dorsal striatum, nucleus accumbens, and lateral ventral tegmental area, and reduces MPA *via* its effects on reward stimulation (Cassilhas et al., 2016). In empirical studies, physical exercise has been shown to improve a range of problems associated with symptoms of addiction, such as withdrawal and mood changes (Chen et al., 2021; Cheng et al., 2021). These results suggest that physical exercise may provide valuable contribution to ameliorating MPA in college students. Therefore, on the issue of MPA, we should pay attention to the role of physical exercise.

Consistent with our hypothesis 1, the results showed that self-control played an indirect role as an independent mediating variable in the association of physical exercise and MPA, and a close association between physical exercise and self-control was found (Yang et al., 2019). When physical exercise decreased, screen use and sedentary behavior increased, the ability to limit and successfully manage their behavior decreased, and the individual’s ability to inhibit and control undesired behavior also decreased (Yang et al., 2019). Exercise was also shown to improve inhibitory, which was explained by better performance on the allocation of attention and larger amplitude of the P3 event-related potential (Xue et al., 2019). In addition, a lack of self-control will lead to behavior changes in college students’ responses to bad emotions, and the risk of MPA will increase accordingly. From a physiological perspective, MPA is associated with inhibitory control areas of the brain (Liu S. et al., 2019), and based on the effect of exercise on prefrontal cortex-dependent executive function, and physical exercise may mitigate addictive behaviors through its effect on inhibitory control (Verburgh et al., 2014). Previous studies have found that exercise can improve the inhibition and control ability deficits of individuals with MPA (Davis et al., 2011). When faced with cognitive tasks, the scores of MPA of individuals doing more exercise were significantly lower (Zhou and Wang, 2022). Thus, increasing self-control and physical exercise may prevent or alleviate MPA.

Consistent with our hypothesis 2, the results showed that rumination played an indirect role as an independent mediating variable in the association of physical exercise with MPA, and found a close association between physical exercise and rumination. Previous studies have found that exercise improves emotional processing (Brand et al., 2018), alleviates negative thinking (Abdollahi et al., 2017), and regular exercise (Lavadera et al., 2020) can reduce rumination by changing the way individuals process and respond to emotions (Bernstein and Mcnally, 2017). Meanwhile, physical exercise linked to memory bias can increase neuroplasticity in the hippocampal circuit (Hamilton and Gotlib, 2008), reducing rumination symptoms (Schnell and Krampe, 2020). In addition, studies have found that rumination mediates the relationship between stress and MPA, suggesting that rumination can predict MPA (Peng et al., 2022). Davis’s cognitive behavioral theory suggests that the distal contributory causes of pathological Internet use (PIU) is stressful life event (i.e., reduced physical exercise) and the proximal contributory causes is maladaptive cognitions (i.e., rumination), which provide a sufficient condition for the formation of PIU (Davis, 2001). Furthermore, self-focused rumination leads an individual to recall more reinforced memories about the Internet, thus maintaining the vicious cycle of MPA (Peng et al., 2022). Thus, it is understandable that physical exercise affects MPA indirectly through rumination.

Consistent with our hypothesis 3, the results showed that psychological distress, as an independent mediating variable, played an indirect role in the association of physical exercise with MPA, and found a close association between physical exercise and rumination. Physiological indicators showed that exercise may be linked to decreased psychological distress by leading to greater diversity in the microbiome (Dalton et al., 2019). Meanwhile, exercise has been shown to increase the brain’s production of endorphins, when the increased release of endorphins can reduce pain or cause euphoria, which in turn can reduce symptoms of depression or anxiety (Li Y. et al., 2021). The sedentary individuals may also be interfered by the hypothalamic–pituitary–adrenal axis and their serum cortisol levels might be altered to cause psychological distress (Uddin et al., 2020). In an exercise intervention study, physical exercise has been proven to be an effective way to improve psychological problems (loneliness, anxiety, lyrical disorders; Mandolesi et al., 2018). In addition, previous studies have acknowledged that psychological distress is an essential catalyst for the development of MPA (Chen et al., 2021). Elhai and Dvorak found that people with depression or anxiety were more likely to use mobile phones and have a high risk of MPA (Elhai et al., 2017). According to the Psychological Decompensation Hypothesis (King and Delfabbro, 2014), when individuals experience negative emotions, such as stress, depression, and anxiety, they are more likely to cope with and alleviate their negative feelings through compensatory mobile phone use. Thus, along with increasing physical exercise, decreasing psychological distress may prevent or alleviate MPA.

In order to offer a more granular understanding of the pathways that associate physical exercise with MPA. The study uses chain mediation models to analyze the four-way association of physical exercise, self-control, rumination, and MPA. Consistent with our hypothesis 4. The results suggest that the association was partially mediated by self-control and rumination. The pathway indicated that physical exercise was sequentially correlated with self-control in the first step and further affected rumination, which was associated with the risk of MPA. The results supported an association between self-control and rumination, that is, an increase in the self-control is associated with a decrease in the rumination. Previous research found that college students with low cognitive control ability may engage in self-focused reflection to reduce the interference in daily life caused by uncontrolled use of mobile phones (Friedman and Miyake, 2017). In the process, if their reflection focuses on the causes and/or consequences of excessive use of mobile phones, rather than measures aimed at reducing their dependence on mobile phones, then the reflection of daily life disruption due to excessive use of mobile phones may promote rumination (Dwyer et al., 2014). In other words, when physical exercise is insufficient, it leads to decreased self-control, increased habitual rumination, and further raised the risk of MPA. Therefore, factors of both self-control and rumination should be considered when designing strategies to reduce MPA through physical exercise.

Consistent with our hypothesis 5, The study analyzes the four-way association of physical exercise, self-control, psychological distress, and MPA using chain mediation models. The results suggest that the association was partially mediated by self-control and psychological distress. The pathway indicated that physical exercise was sequentially correlated with self-control in the first step and further affected psychological distress, which was associated with the risk of MPA. This study supports that self-control is negatively correlated with psychological distress. According to the dual systems model of self-control, the behavior of individuals with low self-control is more easily affected by the impulse system. When encountering negative life events, college students with low level of self-control may be more immersed in negative emotions and more inclined to satisfy the impulse of using smartphones immediately to seek consolation, which leads to MPA (Chen et al., 2021). The mastery hypothesis showed that as exercisers become more confident and gain mastery of their physical skills, they may take this feeling of control and success into their everyday lives and improve their mental health (Mellion, 1985). In addition, previous studies have confirmed that individuals with low self-control had lower timidity in performing inhibitory control tasks, which led to excessive consumption of psychological resources and increased psychological distress (Schnell and Krampe, 2020). Thus, factors of both self-control and psychological distress should be considered when designing strategies aimed at reducing MPA through physical exercise.

Consistent with Hypothesis 6a, loneliness moderates the relationship between self-control and rumination in the chain pathway. Specifically, the indirect effects of physical exercise on MPA through self-control and rumination were buffered by loneliness, with this effect being more substantial for college students with higher levels of loneliness. This result might indicate that loneliness, as a negative personality trait, could have an adverse effect on mental health. Our findings were consistent with the ruminative stress response model: high loneliness, social exclusion, and social isolation would make individuals more likely to reflect on their life and emotional state and then indulge in rumination (Cacioppo et al., 2015). Meanwhile, individuals who feel lonely have particular-cognitive biases and attributional styles. There is evidence that individuals with high loneliness indulge in negative evaluations, stimulate negative cognitive attributions, and lack interpersonal trust (Mann et al., 2017). In addition, college students with low loneliness are more likely to obtain social support and relationships and adjust their cognitive biases and rumination responses in social interaction (Labrague et al., 2021). Therefore, low loneliness can effectively reduce rumination and affect the relationship between self-control and rumination.

Consistent with Hypothesis 6b, loneliness moderates the relationship between self-control and psychological distress in the chain pathway. Specifically, the indirect effects of physical exercise on MPA through self-control and psychological distress were buffered by loneliness. This can be explained by the lack of belonging among college students. Problems experienced during the period of puberty make college students who believe that nobody understands them to experience feelings of loneliness which may cause depression (Erzen and Cikrikci, 2018). In addition, when entering a new environment, individuals in adolescence have imperfect social skills, and social adequacy is insufficient, which leads to loneliness in the social environment and may indirectly trigger the development of depression (Zhang et al., 2014). That is, individuals with high loneliness may be seen as unsociable and more likely to be isolated and ostracized. These negative experiences may increase individuals’ automatic cognitive and emotional responses to stimuli, leading to psychological distress, including depressive symptoms, anxiety, and stress (Labrague et al., 2021). In addition, Houtjes et al. found that loneliness had an independent effect on the course of depression (Houtjes et al., 2014). Therefore, high loneliness will increase the susceptibility of individuals to anxiety, depression, and other negative emotions, and affect the relationship between self-control and psychological distress.

In conclusion, our findings provide theoretical and practical implications for understanding the prevention and alleviation of MPA. On the theoretical level, it provides supporting evidence for the Interaction of Person-Affect-Cognition-Execution (I-PACE) model, the compensatory Internet theory, Salmon’s unifying theory, and the temporal self-regulation theory for physical activity and offers a reinforcement addition to these theories. Our findings confirm the association between physical exercise and MPA and further clarify the underlying mechanisms. These findings may be useful for future research studying the causal association between health and risk-related behaviors and psychological health. In terms of practical implications, the results from our model suggested that physical exercise, self-control, rumination, loneliness, and psychological distress were directly or indirectly associated with MPA. This means that when designing physical exercise programs to improve MPA in college students, incorporating methods to increase self-control, reduce rumination, reduce loneliness, and improve psychological distress might need to be taken into consideration.

### Limitations

Firstly, this study used a cross-sectional design, which cannot provide evidence for causality. Future studies could use randomized controlled trial (RCT) to explore the causal relationship between physical exercise and MPA. In addition, our sample of convenience, limits the extent to which we were able to generalize our results to individuals with the most severe of MPA. Secondly, this study only used self-reported questionnaires as the research object of college students, so there may be social expectation bias. Future studies should use multidimensional scale analysis to collect more objective data from multiple information providers, including parents and peers. Thirdly, this study focused primarily on college students, and more research is needed to explore whether the results apply to other samples, such as adults and adolescents. Despite these limitations, this study reinforces previous research by revealing the mediating and regulating mechanisms between physical exercise and MPA.

## Conclusion

In conclusion, this study expands our understanding of the association and mechanism between physical exercise and MPA. We investigated self-control, rumination, psychological distress as mediators, and loneliness as moderators to explain the relationship between physical exercise and MPA. The results showed that self-control, rumination, and psychological distress partially mediated the relationship between physical exercise and MPA. Physical exercise has an indirect effect on MPA through self-control and rumination, and has an indirect effect on MPA through self-control and psychological distress. Moreover, these effects are more substantial for college students with higher a degree of loneliness. Our findings highlight the importance of enhancing physical exercise and reducing loneliness among interventions to prevent MPA among Chinese college students. In addition, considering that self-control, rumination, and psychological distress play a bridging role in the relationship between physical exercise and MPA, parents and educators should help college students avoid MPA by increasing exercise and reducing loneliness to improve their self-control ability, reduce rumination and psychological distress.

## Data availability statement

The original contributions presented in the study are included in the article/Supplementary material, further inquiries can be directed to the corresponding author.

## Ethics statement

The studies involving human participants were reviewed and approved by Medical Ethics Committee of Sichuan University. The patients/participants provided their written informed consent to participate in this study.

## Author contributions

MZ, JS, SC, XZ, JZ, and XC designed the work and were responsible for the overall development of this study, including the planning of sample collection, data analysis, writing, and polishing of the manuscript. MZ, XZ, and JZ were in charge of data collection and analysis of this study. MZ, JS, and XC were in charge of the main revision for this manuscript. SC and MZ were responsible for revising the manuscript and made a great contribution to the final acceptance of the manuscript. All authors contributed to the article and approved the submitted version.

## Funding

This study was supported by Sichuan University (no. 2021CXC27).

## Conflict of interest

The authors declare that the research was conducted in the absence of any commercial or financial relationships that could be construed as a potential conflict of interest.

## Publisher’s note

All claims expressed in this article are solely those of the authors and do not necessarily represent those of their affiliated organizations, or those of the publisher, the editors and the reviewers. Any product that may be evaluated in this article, or claim that may be made by its manufacturer, is not guaranteed or endorsed by the publisher.
